# Development of peptide nucleic acid-based bead array technology for *Bacillus cereus* detection

**DOI:** 10.1038/s41598-023-38877-1

**Published:** 2023-08-01

**Authors:** Prae Noppakuadrittidej, Ratthaphol Charlermroj, Manlika Makornwattana, Sudtida Kaew-amdee, Rungaroon Waditee-Sirisattha, Tirayut Vilaivan, Thanit Praneenararat, Nitsara Karoonuthaisiri

**Affiliations:** 1 National Center for Genetic Engineering and Biotechnology (BIOTEC), National Science and Technology Development Agency (NSTDA), Khlong Luang, Pathum Thani, Thailand 12120; 2 Department of Chemistry, Faculty of Science, Chulalongkorn University, Phayathai Rd., Pathumwan, Bangkok Thailand 10330; 3 Program in Biotechnology, Faculty of Science, Chulalongkorn University, Phayathai Rd., Pathumwan, Bangkok, Thailand 10330; 4 Department of Microbiology, Faculty of Science, Chulalongkorn University, Phayathai Rd., Pathumwan, Bangkok Thailand 10330; 5 Organic Synthesis Research Unit, Department of Chemistry, Faculty of Science, Chulalongkorn University, Phayathai Rd., Pathumwan, Bangkok Thailand 10330; 6 International Joint Research Center on Food Security, Khlong Luang, Pathum Thani Thailand 12121; 7 Institute for Global Food Security, Queen’s University Belfast, Belfast, UK

**Keywords:** Bioanalytical chemistry, Fluorescent probes, Sensors, Analytical biochemistry, DNA probes, Oligonucleotide probes, Fluorescence spectroscopy

## Abstract

Numerous novel methods to detect foodborne pathogens have been extensively developed to ensure food safety. Among the important foodborne bacteria, *Bacillus cereus* was identified as a pathogen of concern that causes various food illnesses, leading to interest in developing effective detection methods for this pathogen. Although a standard method based on culturing and biochemical confirmative test is available, it is time- and labor-intensive. Alternative PCR-based methods have been developed but lack high-throughput capacity and ease of use. This study, therefore, attempts to develop a robust method for *B. cereus* detection by leveraging the highly specific pyrrolidinyl peptide nucleic acids (PNAs) as probes for a bead array method with multiplex and high-throughput capacity. In this study, PNAs bearing prolyl-2-aminocyclopentanecarboxylic acid (ACPC) backbone with *groEL*, *motB*, and *16S rRNA* sequences were covalently coupled with three sets of fluorescently barcoded beads to detect the three *B. cereus* genes. The developed acpcPNA-based bead array exhibited good selectivity where only signals were detectable in the presence of *B. cereus*, but not for other species. The sensitivity of this acpcPNA-based bead assay in detecting genomic DNA was found to be 0.038, 0.183 and 0.179 ng for *groEL*, *motB* and *16S rRNA*, respectively. This performance was clearly superior to its DNA counterpart, hence confirming much stronger binding strength of acpcPNA over DNA. The robustness of the developed method was further demonstrated by testing artificially spiked milk and pickled mustard greens with minimal interference from food metrices. Hence, this proof-of-concept acpcPNA-based bead array method has been proven to serve as an effective alternative nucleic acid-based method for foodborne pathogens.

## Introduction

*Bacillus cereus* is one of major foodborne pathogens that can be found in a wide range of food products ranging from ready-to-eat foods, fermented foods, and dairy products^1^. Due to its tolerance to extreme pH and temperature, *B. cereus* was commonly found in various stages of food processing^2^, resulting in serious foodborne outbreaks worldwide with an overall prevalence as high as 23.746%. In Europe, it is the second cause of the foodborne outbreak after *Staphylococcus aureus*^3^*.* In the USA, around 63,000 people/year got sick with 0.4% hospitalization rate from the *Bacillus* group^4^.

Rapid and accurate methods enabling timely monitoring and detection of *B. cereus* in food can serve as a key mitigation method in reducing its outbreaks. Moreover, given that not all species of *Bacillus* are pathogenic, it is imperative to be able to identify to the level of *Bacillus* species^5^. While a standard ISO 7932:2004 method is available based on agar plate-based counting protocol^6^, this method is time- and labor-consuming (requiring up to 24-h incubation period at 30 °C with an additional 2–7 days for confirmation assay), and requires trained professionals. Alternative molecular methods such as polymerase chain reaction (PCR)-based techniques have been developed in order to identify *B. cereus*^7,8^. However, most of these PCR-based methods rely on low-resolution gel electrophoresis methods to visualize PCR products, making them difficult for result interpretation. Many advanced PCR-based techniques such as quantitative PCR (qPCR) and multiplex PCR have therefore been developed to overcome these limitations^9–11^, but their multiplex capacity is still limited due to complication from primer dimer formation^12^. In addition, most PCR-based methods depend on DNA as a specific probe, which can be degraded by environmental factors, such as temperature, pH, and enzymes.

To overcome these limitations, this study showcases a detection method for *B. cereus* by combining the advantages of multiplex capacity and ease for result acquisition from bead array technique, and highly specific peptide nucleic acid (PNA) as an alternative probe*.* The bead array platform used in this study is based on a high-throughput, sensitive, and multiplex xMAP technology for different types of targets^13,14^. This method utilizes paramagnetic fluorescently barcoded beads functionalized with carboxyl groups for covalent attachment of nucleophilic ligands. Each bead set can be identified by a red laser, and the signal is measured from a fluorescent reporter (R-phycoerythrin) by a green laser^13,15,16^. Compared to traditional DNA microarray techniques, the bead array technology provides several advantages. First, the throughputness of this technique is clearly greater. This is because it is considered semi-automatic, while the DNA microarray technology is not. Second, owing to its automatic washing step, the bead array technology usually exhibits lower background and higher consistency than does the DNA microarray technology^17^. Lastly, the cost of a detector in the bead array technology is much cheaper than that of the DNA microarray, thus making it more practical. As such, various DNA-based bead array systems have been successfully applied for detecting a wide variety of food contaminations such as food allergens^18^, foodborne pathogens in chicken meat^19^ and four foodborne pathogens including *Salmonella* Typhimurium, *Brucella* spp., *B. cereus*, and *Shigella* spp. in dairy products^20^. Interestingly, the reported method for *B. cereus* detection required elevated temperature at 38 °C to obtain detectable signal, likely due to less stability in DNA-DNA binding compared to PNA-DNA binding. This makes it impractical for actual industrial processing^20^. Moreover, all of these reported bead array methods utilized DNA as a specific probe, whose specificity depends significantly on hybridization temperature and length of probes^21^. We surmised that the use of alternative nucleic acid mimics with better binding affinity to DNA target may improve overall sensing performance.

In this regard, PNA, a nucleic acid mimic in which the phosphodiester backbone has been replaced by pseudopeptide backbone, was selected due to its various advantages such as high thermal stability, high sequence specificity, and high sensitivity for mismatch discrimination^22–24^. Combined with the excellent stability of PNA toward nuclease and proteases, PNA has been developed for many applications including nucleic acid detection^25–27^. The newer types of PNAs, such as conformationally constrained PNAs^28^, were successfully developed to exhibit even better performance than does the original PNA (currently known as aegPNA). For example, Vilaivan and co-workers developed pyrrolidinyl PNA, which consists of nucleobase-modified proline and five-membered ring cyclic *β*-amino acids (named as acpcPNA)^29,30^. This molecular feature avoids degradation under basic conditions from intermolecular cyclization – a fact of which happens readily with aegPNA consisting of α-amino acids^31^. Furthermore, acpcPNA has a more rigid structure than does aegPNA, leading to desirable properties such as better antiparallel selectivity, higher binding affinity, and better discrimination power toward single-base mismatch^32^. As such, acpcPNA has been utilized as a probe in DNA sensing studies involving various platforms^33–37^ such as paper-based sensors, surface plasmon resonance techniques, and electrochemical techniques.

To the best of our knowledge, never before has the Luminex bead array platform been combined with acpcPNA for foodborne pathogen detection. There was only one study that employed a similar concept of combining this Luminex bead array platform and aegPNA to improve detection capacity for detection of the *HER2* oncogene^38^. Our developed method was demonstrated to exhibit good sensing performance with required robustness to detect *B. cereus* spiked in milk and pickled lettuce as models for real food samples. Hence, the developed method shows great promise to be adopted as a practical platform for the detection of *B. cereus* and the concept can be further applied for other pathogens in the future.

## Materials and method

### PNA synthesis

The sequences of acpcPNA probes used in this study are shown in Table 1. The PNA probes were synthesized with acetyl-capped *N*-terminus and the *C*-terminus modified with one lysine unit for conjugating with the carboxyl groups on the paramagnetic beads^39^.

**Table 1:** acpcPNA and specific primer sequences used in this study.

ID	Sequence (5′-3′ for DNA and N-C for PNAs)
*groEL* PNA	Ac-GTAGGAAGCACAG-LysNH_2_
*motB* PNA	Ac-CGAACGTTAAGCC-LysNH_2_
*16S rRNA* PNA	Ac-AACGAGCGCAAC-LysNH_2_
*groEL* forward primer	CTGTAGTTGAAGGT
*groEL* reverse primer	Biotin-CACGAGTTGAGTT
*motB* forward primer	GTGAATGTATATCGA
*motB* reverse primer	Biotin-CTGCATATCCTAC
*16S rRNA* forward primer	Biotin-GTCGTCAGCTCGTGT
*16S rRNA* reverse primer	Biotin-CGATTACTAGCGATTCC

### Primer design

Primer sequences were designed by using Primer3 software (https://www.ncbi.nlm.nih.gov/tools/primer-blast/index.cgi?LINK_LOC=BlastHome) based on National Center for Biotechnology Information (NCBI) GenBank database as shown in Table 1.

### Bacterial strains and media

Bacteria were obtained from Thailand Bioresource Research Center, Thailand (TBRC), American Type Culture Collection, United State of America (ATCC), and Department of Medical Sciences, Thailand (DMST) (Table 2). Except for *Bacillus* spp., all were streaked on a 2xYT agar plate (16 g/L tryptone, 10 g/L yeast extract, 5 g/L sodium chloride, and 15 g/L agar) and incubated at 37 °C for 16–18 h. A single colony of bacteria was inoculated in 10 mL of 2xYT broth (16 g/L tryptone, 10 g/L yeast extract, and 5 g/L sodium chloride) and incubated at 37 °C, 250 rpm for 16–18 h. *Bacillus* spp*.* were streaked on LB agar plates (10 g/L tryptone, 5 g/L yeast extract, 5 g/L sodium chloride, and 15 g/L agar) and incubated at 30 °C for 16–18 h. A single colony of *Bacillus* spp. was inoculated in 10 mL of LB broth (10 g/L tryptone, 5 g/L yeast extract, and 5 g/L sodium chloride) and cultured at 30 °C, 250 rpm for 16–18 h.

**Table 2:** Bacterial strains used in this study.

Bacterial strain	Source
*Bacillus cereus*	TBRC 4973
*B. subtilis*	TBRC 2901
*Escherichia coli*	ATCC 25322
*E. coli* O157: H7	DMST 12743
*Staphylococcus aureus*	ATCC 25923
*Salmonella* Enteritidis	ATCC 13076
*Salmonella* Typhimurium	ATCC 13311

### Genomic DNA extraction

Genomic DNA of each bacterial sample was extracted using a QIAamp®DNA Minikit (#51304, Qiagen) according to the user manual. In brief, bacterial cells were collected from a 1 mL sample by centrifugation at 7500 rpm for 5 min. The pellets were suspended in 180 µL of an enzyme solution (20 mg/mL lysozyme in 20 mM Tris–HCl, 2 mM EDTA, and 1.2% Triton-X 100) and incubated at 37 °C for 1 h. The pellets were treated with 20 µL of proteinase K at 56 °C for 2 h before being treated with 4 µL of RNase A (100 mg/mL) at room temperature for 2 min. The genomic DNA was further purified by a Qiagen mini spin column eluting with 50 µL of sterile water. Concentration and purity of the obtained genomic DNA were determined by UV-spectrophotometry (NanoDrop 8000 spectrophotometer, USA) at 260 and 280 nm.

### Multiplex PCR amplification

Genomic DNA (50 ng) was used as a template in the polymerase chain reaction (PCR) to amplify the target amplicons using biotinylated primers. A PCR master mix solution included 1.25 U of Taq DNA polymerase (#M0273S, Biolabs), 50 mM of KCl, a mixture of three biotin-labeled primer sets, and 0.4 µM of dNTP (#N022, SibEnzyme). 35 cycles of PCR were performed using (1) denaturation at 95 °C for 30 s, (2) primer annealing at 52 °C for 30 s, and (3) DNA extension at 72 °C for 1 min. The biotinylated PCR products were analyzed by gel electrophoresis using 1.5% w/v agarose (#2125, OmniPur) in 0.5 × TBE buffer (44.5 mM Tris, 44.5 mM Boric acid, and 1 mM EDTA) and SYBR®Safe. The images of the agarose gels were taken under UV light (302 nm), with size identification by a DNA ladder marker (100-base pairs, M25, SibEnzyme).

### Development of bead array detection

#### Bead conjugation with acpcPNAs

Three sets of fluorescently barcoded beads (#MC10012, #MC10015, #MC10021, Luminex) were coupled with three acpcPNAs specific to *groEL, motB,* and *16S rRNA* genes. Each bead region (5 × 10^6^ beads/region) was resuspended in 20 µL of 0.1 M MES, pH 4.5 (#M5057, Sigma) and linked with a specific acpcPNA (0.1 nmol) and 2.5 µL of 10 mg/mL 1-ethyl-3-(3-dimethylaminopropyl) carbodiimide hydrochloride, (EDC·HCl, Thermo Fisher Scientific) for 30 min (in the dark). After this period, the second batch of EDC·HCl (2.5 µL of 10 mg/mL) was added to the reaction and incubated for another 30 min. The PNA-loaded beads were washed with 0.02% of Tween-20 (BioBasic inc.) and 0.1% SDS (BioBasic inc.) solution before reconstituted in 20 µL of TE buffer pH 8.0 (10 mM Tris–HCl, 0.1 mM EDTA) and kept at 4 °C in the dark until used.

#### Hybridization and detection

Three acpcPNA-bead regions (2,500 beads/region/reaction, 33 µL each) were mixed together in 1.5 × TMAC (4.5 M TMAC, 0.15% Sarkosyl solution, 75 mM Tris–HCl, and 6 mM EDTA, pH 8.0) and transferred to a 96 non-binding well plate (#655901, Greiner Bio-One). The biotinylated PCR product (10 µL) was added to TE buffer (7 µL), heated at 95 °C for 10 min and placed on ice rapidly for 3 min to maintain the DNA in single stranded form. For the hybridization step, the denatured PCR product solution was added to the bead mixture in the 96 non-binding well plate and incubated at 25 °C with shaking for 1 h before removing any unbound PCR products by washing three times with 100 µL of 1 × TMAC buffer (3 M TMAC, 0.1% Sarkosyl solution, 50 mM Tris–HCl, and 4 mM EDTA, pH 8.0) with the aid of a magnetic separator plate. R-phycoerythrin-labeled streptavidin (25 µL of 10 µg/mL SAPE, #S866, Life technology™, USA.) in 1 × TMAC buffer was added to the plate and incubated in a microplate shaker incubator (Hercuvan Lab systems, USA.) at 25 °C for 15 min. The plate was washed with 100 µL of 1 × TMAC buffer for three times to remove unbound streptavidin before resuspended in 75 µL of MAGPIX Drive fluid (#40-50030, Luminex). Green laser in a Luminex instrument (MAGPIX™, Luminex, USA) was used to measure signal from reporter molecules at 525 nm. Red laser was used to identify specific bead set at 635 nm. Fluorescence signals from SAPE were measured and reported as median fluorescent intensity (MFI), which would be considered as a positive result when its value was at least 2 folds higher than the background or the negative control (distilled water as a template in PCR amplification).

### Specificity, sensitivity, and the limit of detection

To evaluate specificity of the developed method, genomic DNA (50 ng) of six non-target foodborne pathogens (*B. subtilis*, *E. coli*, *E. coli* O157:H7, *Staphylococcus aureus, Salmonella* Enteritidis, and *Salmonella* Typhimurium) were used as templates in the PCR reactions. The PCR products were analyzed with the same PNA bead array using the same protocol as *B. cereus* described above (five replicates).

For sensitivity, different concentrations of genomic DNA (0.002–200 ng) of *B. cereus* were used as templates in PCR reactions and analyzed with the bead array (ten replicates).

The limit of detection (LOD) values were calculated as the concentration of genomic DNA with a signal greater than 2 folds of the background or the negative control (distilled water as a template in PCR amplification). The mean fluorescent intensity (MFI) signal were fitted to the following dose–response curve equation.

$$Y=A+(B/(1+10^{(C-X)} )).$$

Y is a fluorescent intensity from RPE when detecting genomic DNA of pathogen concentration X. whereas A, B, and C are constants from curve fitting.

### Performance comparison between DNA-based and acpcPNA-based bead array methods

Three specific DNA probes (0.1 nmol) with a hexyl spacer with equivalent sequences to those of acpcPNA were coupled with three beads regions (#MC10026, #MC10061, #MC10027, Luminex) via carbodiimide coupling as described in the section about bead conjugation with acpcPNAs. Thereafter, the DNA coupled beads were tested for sensitivity in detecting different concentrations of genomic DNA as described in sensitivity section.

### Detection of *B. cereus* in artificially spiked food samples

To validate the developed method, artificially spiked food samples were tested. Two types of food samples (milk and pickled mustard greens) were collected from local supermarkets (12 samples per sample type). The samples were tested for the absence of *B. cereus* by the International Organization for Standardization 7932 (ISO 7932) method before the spiking experiment. Each *B. cereus*-free food sample (25 g) was homogenized with 225 mL of LB broth in sterile blender bags (#AES400/50G) for 2 min and spiked with *B. cereus* (10 CFU/mL in LB broth) followed by incubation at 30 °C for 16–18 h with 250 rpm shaking. Genomic DNA were extracted from the enriched food samples by a QIAamp®DNA Minikit as described in genomic DNA extraction section. The extracted DNA (2 µL) was used as a template for the PCR amplification and analyzed by the bead array as described above (three replicates).

## Results and discussion

### Design and optimization of acpcPNA-based bead array

**Figure 1:**
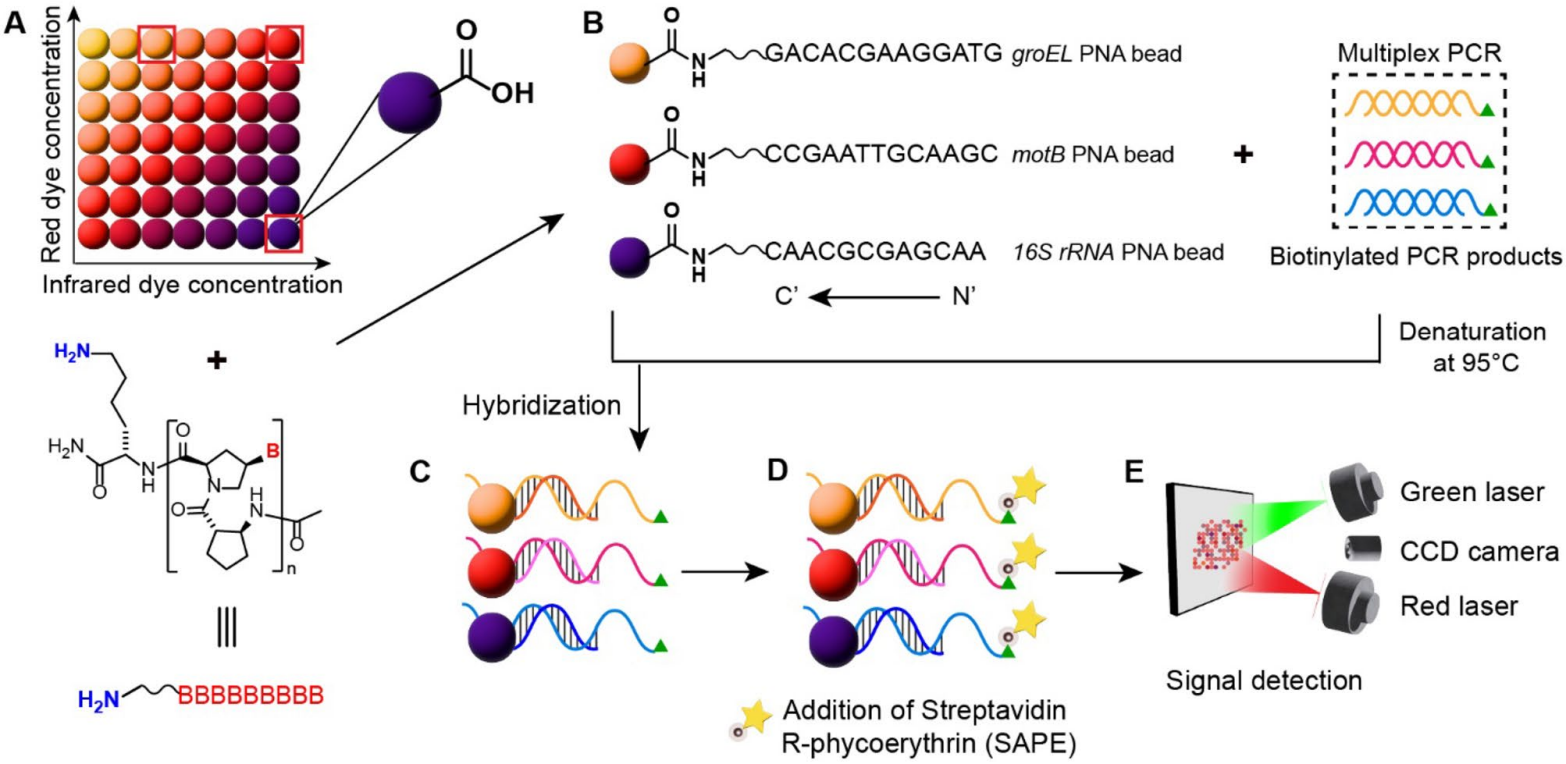
Schematic of acpcPNA-based bead array method. (**A**) A carboxyl group on each fluorescently barcoded paramagnetic bead allows covalent conjugation with the amino group on the acpcPNA molecule. (**B**) Three bead sets coupled with three specific acpcPNAs to *groEL*, *motB* and *16S rRNA* genes were used to detect biotinylated PCR products from a multiplex PCR method. (**C**) Biotinylated single-stranded DNA after denatured at 95 °C was hybridized to acpcPNA-based beads. (**D**) R-phycoerythrin-labeled streptavidin (SAPE) molecules were bound to the biotin tag on the DNA-PNA bead complex. (**E**) A green laser was used to detect fluorescent signal from SAPE and a red laser was used to identify the region of bead set.

To distinguish *B. cereus* from other species in the *Bacillus* groups (*B. anthracis*, *B. cereus*, *B. thuringiensis*, *B. mycoides*, *B. pseudomycoides*, and *B. weihenstephanensis*)*,* chaperone *groEL* and mobility *motB* genes have previously been selected as robust biomarkers through bioinformatic method due to their unique sequences in *B. cereus* among other members of the genus^37^. For internal control, the essential *16S rRNA* gene is selected due to its highly conserved sequence among the bacteria^40^. Indeed, we have previously succeeded using acpcPNA of these genes as a specific probe and internal control in developing a highly specific and sensitive paper-based sensor to detect *B. cereus*^37^. Importantly, while the paper-based platform is theoretically capable of multiplex analysis, the development of a paper-based sensing device with more than three gene targets is generally very tricky. On the other hand, a total of 50 gene targets can be included in one single assay well using bead array technology, thanks to its semi-automatic nature. Therefore, this study aims to develop a proof-of-concept acpcPNA-based bead array technology to empower the multiplex and high-throughput capacity of bead array platform for foodborne pathogen detection with superior specificity. Three fluorescently barcoded bead regions were linked with acpcPNA probes whose sequences match *groEL, motB,* and *16S rRNA* via the coupling between the carboxyl group on the bead and the amino group at the *N*-terminus of the PNA^41^ (Fig. 1A). The bead-immobilized acpcPNA probes were next hybridized with the biotinylated amplicons obtained from multiplex PCR via a strong Watson & Crick base pairing (Fig. 1B,C). This was followed by labeling of the bead-captured biotinylated DNA by an R-phycoerythrin-labeled streptavidin and the fluorescence intensities were measured by dual lasers (Fig. 1D,E).

To evaluate binding efficiency of synthesized acpcPNA sequences to their corresponding complementary DNAs, each type of acpcPNA-bead (*groEL*, *motB* and *16S rRNA*) was separately hybridized with its complementary DNA sequences (Fig. S1). The result indicated that the acpcPNAs were able to bind specifically to their complementary DNAs in all cases. This successful result can be attributed to the conformationally constraint nature of the PNA and the lack of negative charges on the backbone – both of which contributed to the strong binding affinity^32^. In addition, the combination of all three beads and their complementary DNA still showed clear fluorescence signals, albeit with slightly decreased intensities. This finding confirmed that this sensing system has potential for more complex studies.

Thereafter, a series of optimizations was performed to obtain as good performance as possible for hybridization experiments with amplicons, which are much larger in size than the complementary DNA strands. Key parameters to be optimized included hybridization time, concentrations of the reporter molecule (R-phycoerythrin-labeled streptavidin), and concentrations of primers. A range of hybridization times from 15 to 60 min were tested. As shown in Fig. S2, 30-min hybridization gave the highest signal to background ratio for *groEL* gene, while 60-min hybridization was the best for *motB* and *16S rRNA* genes. Thus, 60 min of hybridization time was chosen. Also, Fig. S3 suggested that 10 µg/mL of the reporter molecule gave the best results, where the signal-to-background ratios were at 8.41, 4.06, and 2.44 for *groEL*, *motB*, and *16S rRNA* genes, respectively. Lastly, since complication can arise from multiplex PCR, primer concentrations are also a key factor to be optimized. This was done by first focusing on the control gene (*16S rRNA)* primer as it likely dominates the amplification due to its high abundance in the genomic DNA template. Preliminary experiment suggested that 50 nM is the smallest concentration of the *16S rRNA* primer that can still provide appreciable signal. Also, another separate experiment established that 1:2 amount ratio of *groEL*:*motB* primers provided the greatest signal intensity for simultaneous amplification using these two genes. Thereafter, we put these preliminary data together into the final optimization for multiplex PCR as shown in Fig. S4, where both the concentration of the *16S rRNA* primer (at 50 nM), and the amount ratio of the *groEL* and *motB* primers (1:2) were held constant. That is, only the concentrations of *groEL* and *motB* primers were varied. The results suggested that, excluding the lowest concentrations tested, other conditions did not give significantly different outcomes. Hence, the second lowest concentrations of primers seemed to be the most logical choice. However, this condition (300:600:50 nM of *groEL, motB, and 16S rRNA* primers, respectively) could not result in appreciable amplifications in other tested bacterial species. Thus, the concentrations of 400:800:50 nM of *groEL, motB, and 16S rRNA* primers, respectively, were instead selected for subsequent studies.

### Evaluation of sensing performance

**Figure 2:**
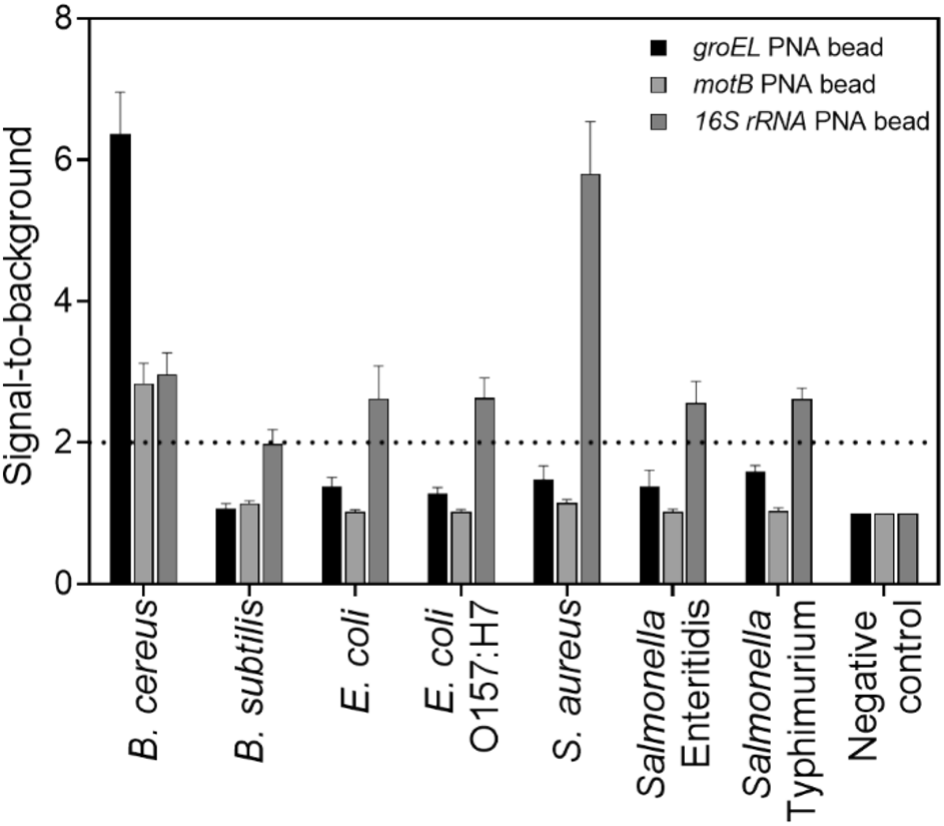
Specificity of the acpcPNA-based bead array detection against *B. cereus*, *B. subtilis*, *E. coli*, *E. coli* O157:H7, *S. aureus*, *Salmonella* Enteritidis, and *Salmonella* Typhimurium. Genomic DNA sample (50 ng) of each species was tested. Each data group was plotted as an average of five-replicates with an error bar that indicated a standard deviation. The dotted line represents a cut-off value which is two times of the intensity from negative control. The negative control had water as a template for amplification.

Specificity of the developed method was evaluated against seven relevant foodborne species namely *B. cereus*, *B. subtilis*, *Escherichia coli*, *E. coli* O157:H7, *Staphylococcus aureus*, *Salmonella* Enteritidis, and *Salmonella* Typhimurium. In each case, the isolated genomic DNA was used as a template for the multiplex PCR amplification with biotin tagging to all three target genes and tested by the acpcPNA-based bead array. In the case of *B. cereus* detection, both *groEL* and *motB* beads exhibited positive results with the highest signal from the *groEL* bead at approximately 7 folds of signal-to-background ratio (Fig. 2). The signals from *groEL* and *motB* beads for other tested bacteria were negative, while the internal positive control from the *16S rRNA* beads were positive in all bacteria species, thus confirming that this gene can be employed as a robust internal control for this sensing platform.

Overall, while the signals obtained from the detection of longer DNA amplicons (200–310 bp) were lower than those obtained from short synthetic DNA oligonucleotides (Fig. S1), the results are sufficient for unambiguous identification of *B. cereus*. This decrease in signal intensity is unsurprising because amplicons used herein are 200–310 bp, which are much longer than probe sequences on the solid support (12–15 bp). Given the chance of forming secondary structures from DNA in this length^16,42^, the results are considered to be decent. Moreover, the lower signal could be from the complication of multiplex PCR amplicons. The increased number of primer sets for multiple targets within a single reaction could generate spurious amplification products due to formation of primer dimer^43^.

**Figure 3:**
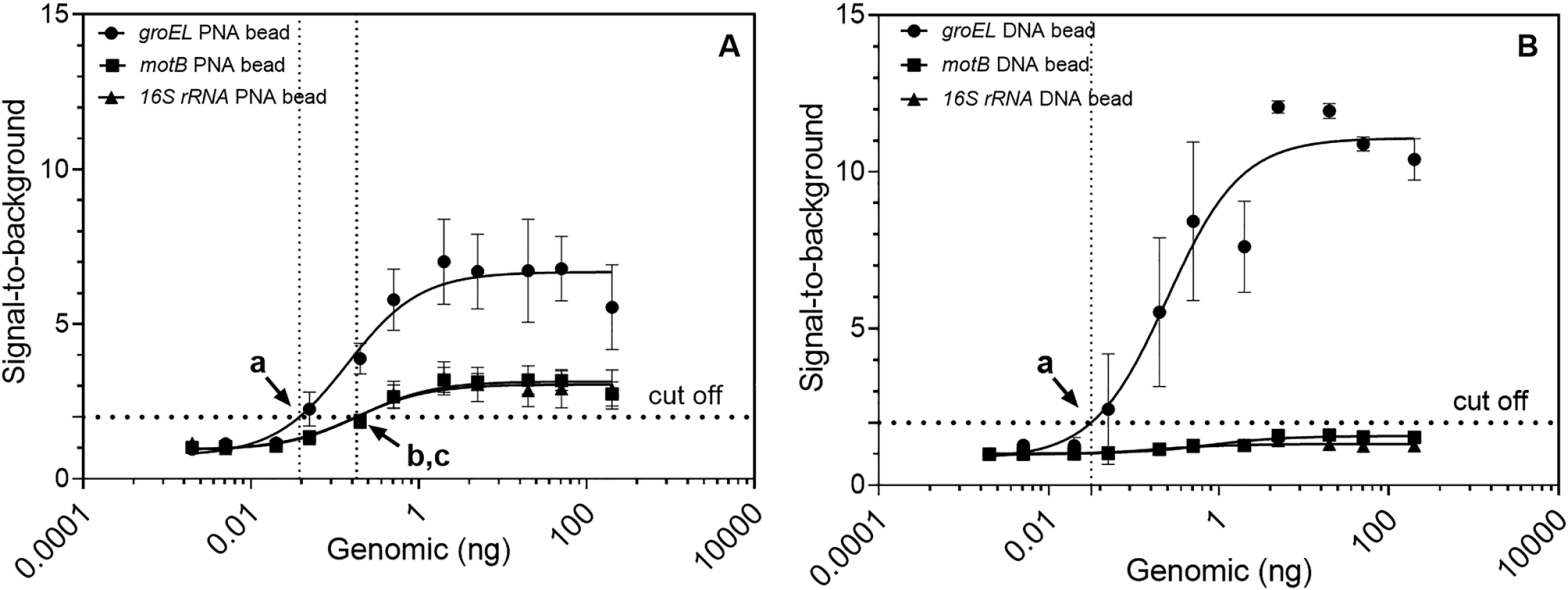
Responses of (**A**) acpcPNA-based arrays (LOD = 0.038 ng (a), 0.183 ng (b), and 0.179 ng (c) of genomic DNA for *groEL*, *motB*, and *16S rRNA* PNA beads, respectively), and (**B**) DNA-based bead arrays (LOD = 0.032 ng (a) for *groEL* DNA bead). Each data point was plotted as an average of ten replicates with an error bar that indicated a standard deviation. The dotted line at the bottom of the graph represents a cut-off value which is two times the intensity from the negative control.

The limit of detection (LOD) of this method was evaluated for all genes used in detecting known genomic DNA concentrations (Fig. 3A). A clear increase of fluorescent intensities was shown around 0.1 ng, where signal started to rise in a linear fashion up to around 1 ng before reaching a plateau^16^. The *groEL* PNA bead was able to detect as low as 0.038 ng of genomic DNA, while the *motB* and *16S rRNA* beads gave LOD values of 0.183 and 0.179 ng of genomic DNA, respectively. The obtained LOD values are less sensitive than our previous paper-based sensor^37^ which could be due to the use of multiplex PCR which is known to be more complicated to develop and often less sensitive than conventional PCR^44^. However, the multiplex capacity of this developed method and the ease of a single step multiplex PCR offer an important advancement of the detection method as it can further include detection of other important genes such as emetic toxin genes from *B. cereus* strains^42^ and specific genes for other bacteria. Importantly, the superior performance of acpcPNA over DNA was also proven by evaluating the sensing performance of DNA probes having the same sequences as the acpcPNA probes used in this study (Fig. 3B). Interestingly, while the LOD of *groEL* DNA bead was comparable to its acpcPNA counterpart (0.032 ng vs 0.038 ng respectively), the signal from the *groEL* DNA bead seemed to reach somewhat higher plateau, i.e., higher signal-to-background ratio, at much higher amount of genomic DNA sample (~ 30 ng) than did the acpcPNA bead. Nonetheless, the *motB* and *16S rRNA* DNA beads could not detect genomic DNA in any amounts of genomic DNA tested (Fig. 3B**)**. This could be explained by the low stability of the duplexes formed between the relatively short DNA probes and the DNA target. The results confirm the superior performance of acpcPNA over DNA probes in terms of robustness in the multiplex assay.

### Assay performance in real matrices

**Figure 4:**
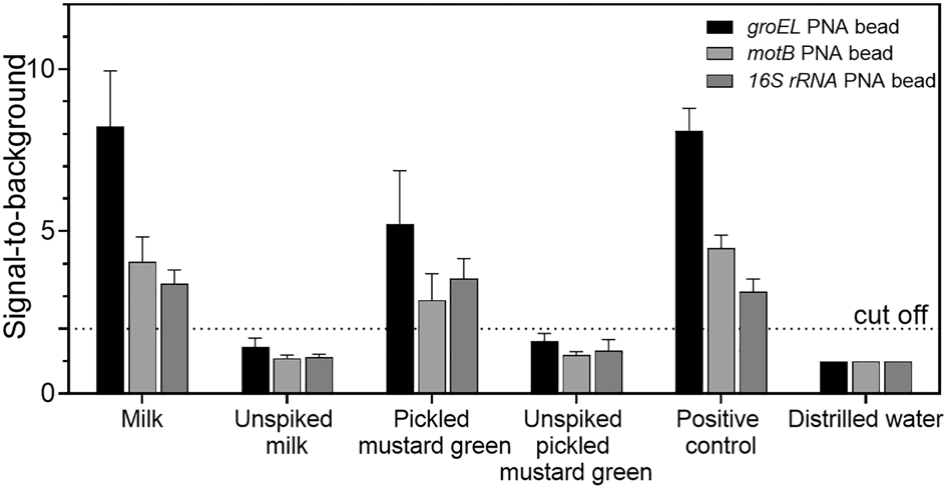
The acpcPNA-based bead array used to detect *B. cereus* in artificially spiked food samples. *B. cereus* (10 CFU/mL) was spiked into milk, pickled mustard greens, and positive control (culture media). Each data group was plotted as an average of 12 samples with an error bar that indicated a standard deviation. The dotted line represents a cut-off value which is two times of the intensity from the negative control (water as a template in the PCR amplification).

*B. cereus* outbreaks have been reported in various foods, such as starchy foods and rice, raw and processed vegetables, bread, milk and dairy products, meat products, and ready-to-eat foods^2,3^. To validate the developed method, two types of food matrices namely milk and pickled mustard greens were selected to represent dairy products and ready-to-eat foods. The food samples were confirmed to be free of *B. cereus* by the ISO 7932 method before spiking experiments. The *B. cereus*-free milk and pickled mustard greens (n = 12 per each food matrix) were spiked with 10 CFU/g of *B. cereus*. This figure is lower than the maximum acceptable number of *B. cereus* (10^2^ CFU/mL and 50 CFU/g for children under the age of 6 months) according to the Codex Alimentarius Commission of the Food and Agriculture Organization of the United Nations (FAO) and the World Health Organization (WHO)^45^. Thereafter, the spiked samples were enriched by incubation at 30 °C overnight, followed by genomic DNA extraction. The biotinylated PCR products were prepared by multiplex PCR under the optimal condition obtained previously, and the amounts of the DNA target sequence for the three genes were measured by the acpcPNA-based bead array. The culture media with 10 CFU/g of *B. cereus* was used as a positive control. There were three types of negative controls in this study to cover all possibilities including the use of distilled water as a template for PCR amplification, and the use of unspiked matrices for DNA purification prior to PCR amplification. Gratifyingly, the developed assay was able to identify three specific genes of *B. cereus* with little interference of the food matrix (Fig. 4). The *groEL* bead again provided the highest signal in both matrices. The lower signals obtained from pickled mustard greens are likely due to the presence of various inhibitors such as enzymes, polysaccharides, proteins, and salt, all of which could interfere with genomic DNA extraction and subsequent PCR amplification^9^. Nevertheless, the data from real food matrices clearly demonstrated that the developed acpcPNA-based bead array can be adopted as an alternative method for specific and multiplex detection for foodborne pathogens.

## Conclusion

In this study, we successfully developed a multiplex acpcPNA-based bead array technique to identify *Bacillus cereus* with good specificity, low limit of detection, and high-throughput capacity. The total assay time of 4 h, including multiplex PCR amplification, is shorter than the culture-based ISO method which requires at least 72 h to identify *B. cereus*. The validation of the developed method with representative real food matrices indicates that our method can detect the prevalent *B. cereus* pathogen accurately and its practicality can further be explored for food safety support in industrial processes.

## Supplementary Information

Supplementary Figures.

## Data Availability

The datasets analyzed during the current study are available in the GenBank repository, [*groEL* Gene ID: 72447092; *motB* Gene ID: 72451176; *16S rRNA* Nucleotide ID: NR_074540.1].
