# Supplementary material for: Development of peptide nucleic acid-based bead array technology for Bacillus cereus detection
Source: Sci Rep. 2023 Aug 1;13:12482. doi: 10.1038/s41598-023-38877-1 (PMC10393979; doi:10.1038/s41598-023-38877-1)
Supplement: Supplementary file 1 — Supplementary Figures. [file 41598_2023_38877_MOESM1_ESM.pdf]

Supplementary Information

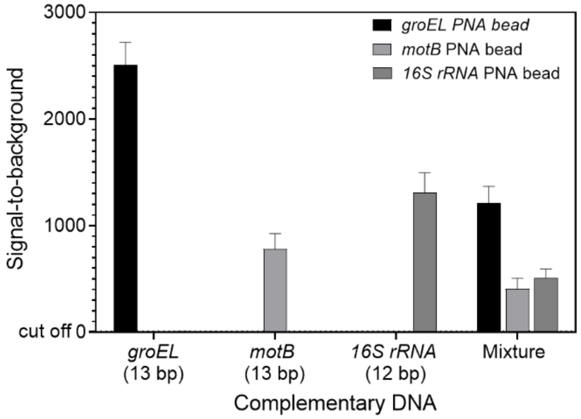

Fig. S1. Evaluation of binding efficiency between PNA probes on bead with their complementary DNA.

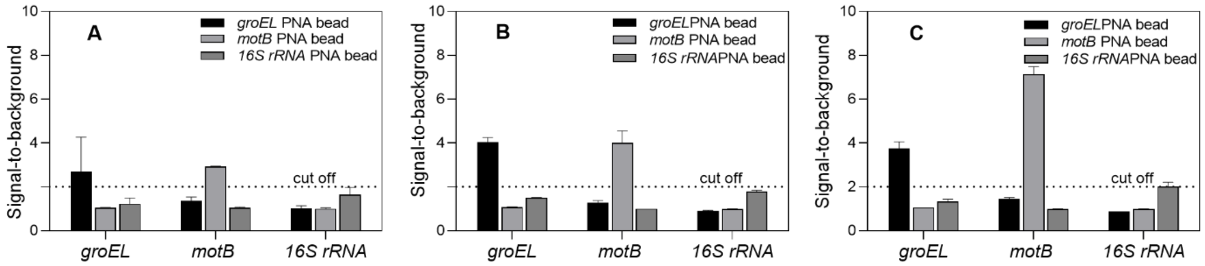

Fig. S2. Optimization of hybridization time for the detection of three target genes: A) 15 minutes; B) 30 minutes; and C) 60 minutes. The dotted line represents a cut-off value, which is two times of the intensity from the negative control. Each data point was plotted as an average of three replicates with an error bar that indicated a standard deviation.

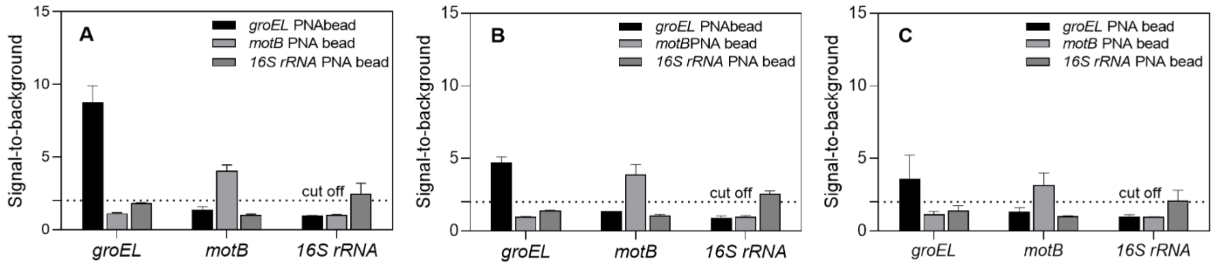

Fig. S3. Optimization of R-phycoerythrin-labeled streptavidin (SAPE) concentrations for the detection of three target genes: A) 10 µg/mL; B) 20 µg/mL, and C) 40 µg/mL of SAPE concentrations. The dotted line represents a cut-off value which is two times of the intensity from the negative control. Each data point was plotted as an average of three replicates with an error bar that indicated a standard deviation.

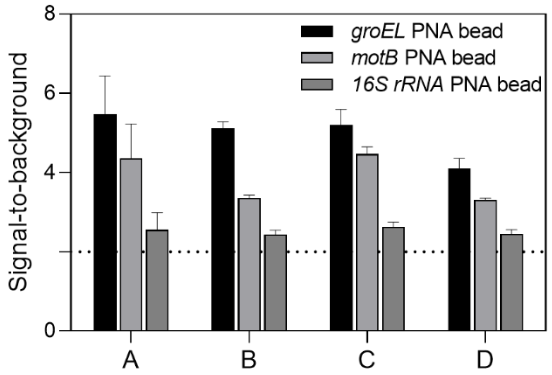

Fig. S4. Optimization of primer concentration of multiplex PCR products in a range of 50 nM to 1000 nM. A.) Primer concentration at *groEL* 500 nM, *motB* 1000 nM, and *16S rRNA* 50 nM. B.) Primer concentration at *groEL* 400 nM, *motB* 800 nM, and *16S rRNA* 50 nM. C.) Primer concentration at *groEL* 300 nM, *motB* 600 nM, and *16S rRNA* 50 nM. D.) Primer concentration at *groEL* 200 nM, *motB* 400 nM, and *16S rRNA* 50 nM. The dotted line at the bottom of the graph represents a cut-off value which is two times of the intensity from the negative control. Each data point was plotted as an average of three replicates with an error bar that indicated a standard deviation.
